# Non-Linear Relationship between Serum 25-Hydroxyvitamin D and Hemoglobin in Korean Females: The Korean National Health and Nutrition Examination Survey 2010–2011

**DOI:** 10.1371/journal.pone.0072605

**Published:** 2013-08-28

**Authors:** Seung Seok Han, Myounghee Kim, Ho Kim, Su Mi Lee, Yun Jung Oh, Jung Pyo Lee, Sejoong Kim, Kwon Wook Joo, Chun Soo Lim, Yon Su Kim, Dong Ki Kim

**Affiliations:** 1 Department of Internal Medicine, Seoul National University College of Medicine, Seoul, Korea; 2 Department of Dental Hygiene, College of Health Science, Eulji University, Gyeonggi-do, Korea; 3 Department of Epidemiology and Biostatistics, School of Public Health, Seoul National University, Seoul, Korea; 4 Department of Internal Medicine, Seoul National University Boramae Medical Center, Seoul, Korea; 5 Department of Internal Medicine, Seoul National University Bundang Hospital, Gyeonggi-do, Korea; Wayne State University School of Medicine, United States of America

## Abstract

**Background:**

Anemia and vitamin D deficiency are both important health issues; however, the nature of the association between vitamin D and either hemoglobin or anemia remains unresolved in the general population.

**Methods:**

Data on 11,206 adults were obtained from the fifth Korean National Health and Nutritional Examination Survey. A generalized additive model was used to examine the threshold level for relationship between serum 25-hydroxyvitamin D [25(OH)D] and hemoglobin levels. A multivariate logistic regression for anemia was conducted according to 25(OH)D quintiles. All analyses were stratified according to sex and menstrual status.

**Results:**

The generalized additive model confirmed a threshold 25(OH)D level of 26.4 ng/mL (male, 27.4 ng/mL; premenopausal females, 11.8 ng/mL; postmenopausal females, 13.4 ng/mL). The threshold level affected the pattern of association between 25(OH)D and anemia risk: the odds ratio of the 1^st^ quintile but not the 2^nd^, 3^rd^, and 4^th^ quintiles were significantly different from the 5^th^ quintile in both premenopausal and postmenopausal females, however there was no obvious trend in males.

**Conclusions:**

This population-based study demonstrated a non-linear relationship with a threshold effect between serum 25(OH)D and hemoglobin levels in females. Further interventional studies are warranted to determine whether the appropriate level of hemoglobin can be achieved by the correction of vitamin D deficiency.

## Introduction

Anemia is a major global health concern due to its high prevalence and association with substantial morbidity and mortality [1], [2]. Despite its importance in public health and the consistent implementation of strategies to control anemia, its prevalence remains relatively unchanged [3]. This is attributable to several reasons: (1) the multifactorial and interactive nature of the etiologies makes anemia difficult to prevent or treat; (2) the comorbidities related to anemia, such as chronic kidney disease, have an increasing frequency; and (3) the distribution of the population around the world is shifted toward the elderly, who have a high prevalence of anemia [4], [5], [6]. In addition, unestablished etiologies may contribute to the sustained high prevalence of the disease. Consequently, efforts to identify less well-known, but potentially modifiable, factors associated with anemia will be required to reduce the large burden of anemia.

Vitamin D deficiency is also an important issue in public health because it is associated with a wide range of illnesses and chronic conditions, such as osteoporosis, cancer, metabolic syndrome, and cardiovascular disease [7]. Recently, the role of vitamin D in erythropoiesis has been suggested, but data are limited in the general population [8], [9], [10], [11], [12], [13], [14], [15]. Indeed, most studies regarding the association between vitamin D and anemia have been conducted in diseased individuals prone to anemia, including patients with chronic kidney disease, end-stage renal disease, congestive heart failure, and the elderly [8], [9], [10], [11], [12], [13]. In addition, previous studies have not considered a possible non-linear relationship between vitamin D and hemoglobin levels [8], [9], [10], [11], [12], [13], [14], [15]. Recent large-scale studies have demonstrated that the risks of various clinical conditions and mortality show a non-linear relationship with serum 25-hydroxyvitamin D [25(OH)D] levels, and reach a plateau above certain levels of serum 25(OH)D [16], [17], [18], [19], [20]. In this regard, a threshold level of serum 25(OH)D associated with changes in hemoglobin levels may exist, but no previous studies have considered this issue. In the present study, we aimed to verify the relationship in a representative Korean adult population using a data set from the Korean National Health and Nutritional Examination Survey (KNHANES).

## Methods

### Ethics Statement

This investigation was conducted according to the principles expressed in the Declaration of Helsinki. All participants engaged voluntarily and signed informed consent forms. The institutional review board at the Korea Centers for Disease Control and Prevention approved the survey of the study population (nos. 2010-02CON-21-C, 2011-02CON-06-C).

### Study Population

Data were obtained from the first and second years (2010–2011) of the KNHANES V, which was conducted nationwide by the Korea Centers for Disease Control and Prevention. KNHANES used a rolling sampling design that involved a complex, stratified, multistage, probability cluster survey of a representative sample of the non-institutionalized civilian population in South Korea. The survey consisted of health interviews, examinations, including laboratory tests, and a nutritional survey. For the KNHANES 2010–2011, 384 sampling units were randomly selected from the primary sampling units encompassing the target population in South Korea, with 20 households selected from each primary sampling unit to yield 3840 households each year, totaling 7680 households in 2010 and 2011. A total of 10,938 and 10,589 subjects were sampled in 2010 and 2011, respectively. In total, 8,958 (81.9% in 2010) and 8,518 (80.4% in 2011) subjects participated in the survey each year. The cross-sectional analysis presented here was restricted to 11,685 subjects ≥20 years of age for whom serum 25(OH)D and hemoglobin levels were available. Furthermore, we excluded subjects with a self-reported history of all types of tumor including malignancy (n = 369), liver cirrhosis (n = 33), and delivery within 1 year (n = 77). A total of 11,206 subjects were analyzed in the present study.

### Study Variables

Sociodemographic variables, including age, sex, smoking status, socioeconomic status such as income and educational level, and menstrual status were collected during the health interview. Regular exercise was defined as regular moderate exercise for ≥30 minutes per session more than five times per week or intense exercise for ≥20 minutes per session more than three times per week. Moderate exercise was defined as an activity that leaves the subject somewhat breathless, such as badminton, table tennis, slow swimming, or volleyball. Intense exercise was defined as an activity that causes the subject to be out of breath, such as climbing, basketball, football, squash, or running. Patients reported whether they had been diagnosed with hypertension, diabetes mellitus, or cardiovascular disease by a medical doctor. Weight (kg) and height (cm) were measured in subjects wearing only a gown without shoes. The body mass index was calculated as [weight (kg)/height (m^2^)]. The daily iron intake from food was assessed using a 24-hour recall method.

Blood samples were collected during the fasting state for the health examination surveys. After collection, the samples were promptly refrigerated and transported to the designated central laboratory (NeoDin Medical Institute, Seoul, Korea). Serum 25(OH)D levels were measured using a radioimmunoassay kit (DiaSorin Inc., Stillwater, MN, USA) with a 1470 WIZARD gamma–counter (PerkinElmer, Finland). Serum hemoglobin levels were measured using an SLS hemoglobin (NoCyanide) method with the XE-2100D (Sysmex, Tokyo, Japan). Anemia was defined as serum hemoglobin <13 g/dL in men and <12 g/dL in women according to the World Health Organization criteria [21]. Other blood tests were also performed to evaluate the levels of iron, total iron-binding capacity, cholesterol, triglyceride, and creatinine with a Hitachi Automatic Analyzer 7600 (Hitachi, Tokyo, Japan). The level of serum ferritin was measured by a 1470 WIZARD gamma–counter (PerkinElmer, Finland). The estimated glomerular filtration rate (mL/min/1.73 m^2^) was calculated using the Modification of Diet in Renal Disease equation [22] and the validated coefficient for Korean subjects [23] as follows: 107.904×serum creatinine (mg/dL)^−1.009^×age (years)^−0.02^ (×0.667, if female). The seasons of blood collection were classified as spring (March to May), summer (June to August), autumn (September to November), and winter (December, January, and February).

### Statistical Analysis

All of the analyses and calculations were performed using SAS version 9.2 (SAS Institute, Cary, NC, USA) and R software version 2.15.1 (The Comprehensive R Archive Network: http://cran.r-project.org). In all the analyses, the complex sampling and survey sample weights of the KNHANES were used. The data are presented as the weighted arithmetic means [standard error (SE)] for continuous variables and the weighted proportions (SE) for categorical variables. Based on variable distribution using histograms, the levels of ferritin, cholesterol, triglyceride, and the daily iron intake were non-normally distributed; therefore, these variables were natural log transformed, and the weighted geometric means (SE) were calculated. The baseline characteristics between anemia and non-anemia groups were compared using the generalized linear model for continuous variables and the Pearson’s chi-squared test for categorical variables, respectively.

The lowess (locally weighted smoothing) regression curve on a scatter plot between serum 25(OH)D and hemoglobin levels was used for predicting the simple relationship of both variables. The correlation coefficient (*r*) was measure using the Pearson’s correlation method. Then, generalized additive models (GAMs) for Gaussian distributions were adapted to visualize the associations between serum 25(OH)D and hemoglobin levels after adjustment for potential confounders, including age, sex, smoking, exercise, hypertension, diabetes mellitus, cardiovascular disease, body mass index, season, ferritin, iron, total iron-binding capacity, cholesterol, triglyceride, estimated glomerular filtration rate, and daily iron intake [24]. When a non-linear relationship was observed, piecewise linear regression models were employed to estimate the threshold point of the serum 25(OH)D level related to the serum hemoglobin level [25]. We used Akaike’s information criterion (AIC) as a primary measure of model fit. In AIC, lower scores within the data set indicate a better model fit [26]. A 25(OH)D threshold point was chosen based on the best fit determined by AIC among the models, with 25(OH)D threshold levels that differed in increments of 0.2. We then plotted the threshold level on the GAM model.

A logistic regression analysis was used to further examine the risk of anemia according to the quintiles of serum 25(OH)D levels. The effects of the logistic regression model are shown as odds ratios (ORs) and 95% confidence intervals (CIs). For this analysis, ORs were adjusted for the same covariates as the GAM model. Interactions were tested by adding a product term for 25(OH)D and each covariate. Predicted probability of anemia was calculated from the multivariate logistic regression model. We showed curves of predicted probability and 95% confidence intervals using the lowess regression. The GAM and logistic regression models were also conducted after stratification by sex and menstrual status. A *P* value of less than 0.05 was considered significant.

## Results

### Baseline Characteristics

The mean age of the subjects was 45 years. The mean serum 25(OH)D level was 17.5 ng/mL. Approximately 70.6% of subjects exhibited vitamin D deficiency (<20 ng/mL): 64.2% of males and 77.3% of females. The distribution of the seasons of blood sampling was almost even across the entire study period. Anemia was identified in 7.9% of the study subjects: males, 2.4%; premenopausal females, 15.5%; postmenopausal females, 10.7%. A total of 0.5% of the subjects had an estimated glomerular filtration rate less than 60 mL/min/1.73 m^2^. Other demographic and laboratory findings are presented in Table 1.

**Table 1:** Baseline characteristics of the study subjects.

	Total (n = 11,206)	No anemia (n = 10,196)	Anemia (n = 1,010)	*P* *
Age (years)†	45.0 (0.3)	44.8 (0.3)	48.0 (0.8)	<0.001
Female (%)	49.4 (0.5)	46.4 (0.5)	84.4 (1.6)	<0.001
Smoker (%)	27.4 (0.6)	29.1(0.6)	8.2 (1.1)	<0.001
Income				0.036
1^st^ quartile (%)	26.3 (0.8)	26.0 (0.8)	29.4 (2.0)	
2^nd^ quartile (%)	25.4 (0.7)	25.3 (0.7)	27.0 (1.6)	
3^rd^ quartile (%)	25.2 (0.7)	25.3 (0.7)	24.6 (1.7)	
4^th^ quartile (%)	23.0 (0.8)	23.4 (0.8)	19.0 (1.5)	
Educational level				0.009
Less than high school (%)	28.4 (0.8)	28.0 (0.8)	33.3 (2.0)	
High school (%)	27.8 (0.7)	38.0 (0.7)	36.0 (1.9)	
College and more (%)	33.7 (0.8)	34.0 (0.9)	30.7 (1.8)	
Regular exercise (%)	21.2 (0.6)	21.5 (0.6)	18.7 (1.7)	0.109
Hypertension (%)	16.5 (0.5)	16.3 (0.5)	18.8 (1.4)	0.062
Diabetes mellitus (%)	6.2 (0.3)	6.0 (0.3)	8.3 (1.0)	0.013
Cardiovascular disease (%)	2.9 (0.2)	2.8 (0.2)	4.3 (0.7)	0.017
Body mass index (kg/m^2^)†	23.7 (0.1)	23.8 (0.1)	22.5 (0.1)	<0.001
Season of blood sample collection				0.074
Spring (%)	23.9 (2.4)	23.9 (2.5)	23.5 (2.8)	
Summer (%)	26.6 (2.6)	26.3 (2.6)	30.0 (3.3)	
Autumn (%)	24.3 (2.4)	24.6 (2.5)	20.8 (2.5)	
Winter (%)	25.2 (2.5)	25.2 (2.5)	25.6 (3.0)	
Laboratory finding				
Hemoglobin (g/dL)†	14.2 (0.1)	14.5 (0.1)	11.1 (0.1)	<0.001
Ferritin (ng/mL)‡	56.6 (1.0)	63.5 (1.0)	14.7 (1.0)	<0.001
Iron (µmol/L)†	115.7 (0.7)	119.9 (0.7)	66.6 (1.8)	<0.001
TIBC (µmol/L)†	316.2 (0.6)	313.0 (0.6)	352.4 (3.2)	<0.001
25-hydroxyvitamin D (ng/mL)†	17.5 (0.2)	17.6 (0.2)	16.0 (0.3)	<0.001
Cholesterol (mg/dL)‡	184.7 (1.0)	185.7 (1.0)	173.8 (1.0)	<0.001
Triglyceride (mg/dL)‡	109.3 (1.0)	111.3 (1.0)	88.8 (1.0)	<0.001
Estimated GFR (mL/min/1.73 m^2^)†	101.6 (0.3)	101.8 (0.3)	98.9 (0.8)	<0.001
Daily iron intake from food (mg/day)‡	12.9 (1.0)	2.6 (0.1)	2.4 (0.1)	<0.001

* Statistical differences between anemia and non-anemia groups are calculated.

† Arithmetic mean (standard error).

‡ Geometric mean (standard error).

Abbreviations: TIBC, total iron-binding capacity; GFR, glomerular filtration rate.

### Relationship between Serum 25(OH)D and Hemoglobin Levels

**Figure 1:**
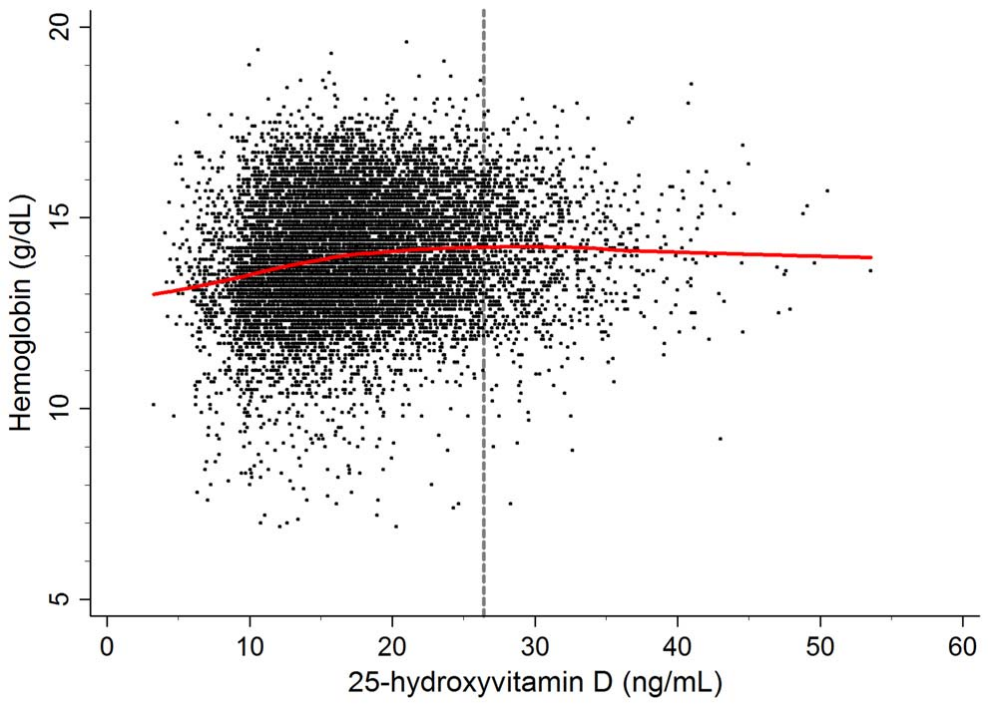
Scatter plot with the lowess regression curve between serum 25-hydroxyvitamin D and hemoglobin levels.

**Figure 2:**
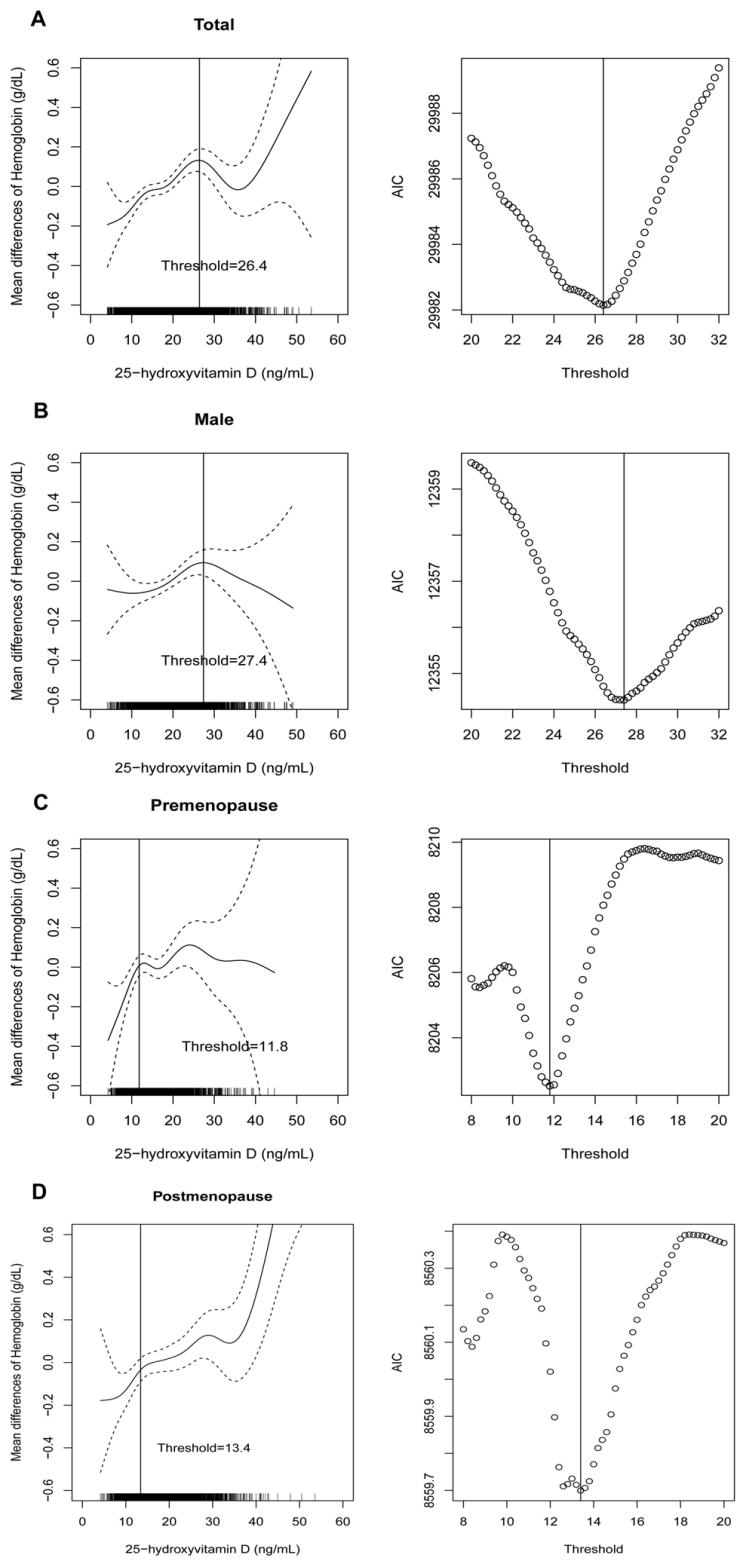
Spline curve (solid line) with 95% confidence intervals (two dashed lines) using generalized additive model for Gaussian distribution and process of determining the threshold level for an association using Akaike’s information criterion. Total subjects (A), males (B), premenopausal females (C), and postmenopausal females (D). AIC, Akaike’s information criterion.

Figure 1 shows a scatter plot between serum 25(OH)D and hemoglobin levels to evaluate the distributions of these variables. As shown in the lowess line in figure 1, the relationship between serum 25(OH)D and hemoglobin levels appeared to be smoothly positive (*r* = 0.140, *P*<0.001); males, *r* = −0.047 (*P = *0.001); premenopausal females, *r* = 0.063 (*P*<0.001); postmenopausal females, *r* = 0.034 (*P = *0.055). From the above observation, we hypothesized that there would be a threshold for an association between the serum 25(OH)D and hemoglobin levels that was attributable to a non-linear relationship between them. Subsequently, we conducted a GAM adjusted for confounding variables in a stepwise manner to further investigate the non-linear relationship between serum 25(OH)D and hemoglobin levels and estimate the 25(OH)D threshold below which serum hemoglobin levels decreased rapidly. In each model, we used AIC as the primary measure of model fit. The lowest AIC level was measured in the model adjusted for all confounding variables, including age, sex, smoking, exercise, hypertension, diabetes mellitus, cardiovascular disease, body mass index, season, ferritin, iron, total iron-binding capacity, cholesterol, triglyceride, estimated glomerular filtration rate, and daily iron intake. Figure 2 shows the GAM plot used to identify the threshold level of 25(OH)D that predicts changes in hemoglobin levels. After comparing AICs among the piecewise linear regression models, 26.4 ng/mL of serum 25(OH)D had the lowest AIC value (Figure 2A): the difference from the mean serum hemoglobin began to increase as the serum 25(OH)D decreased below the threshold level of 26.4 ng/mL. After stratifying the data according to sex and menstrual status, the threshold levels were as follows: males, 27.4 ng/mL; premenopausal females, 11.8 ng/mL; postmenopausal females, 13.4 ng/mL (Figure 2B–2D).

### Risk of Anemia according to the Serum 25(OH)D Levels

**Figure 3:**
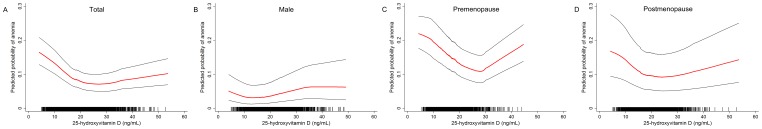
Predicted probability of anemia according to the serum 25-hydroxyvitamin D levels. Total subjects (A), males (B), premenopausal females (C), and postmenopausal females (D). Red and black lines represent predicted probability of anemia and 95% confidence interval, respectively.

We further evaluated the risk of anemia, defined as <13 g/dL in males and <12 g/dL in females, according to quintiles of the serum 25(OH)D level. In univariate analyses, the lower quintiles of serum 25(OH)D had a greater OR for anemia compared with the highest quintile, as follows: 1^st^ quintile, 2.124 (1.624–2.780) (*P*<0.001); 2^nd^ quintile, 1.472 (1.114–1.945) (*P* = 0.007); 3^rd^ quintile, 1.275 (0.954–1.705) (*P* = 0.101); and 4^th^ quintile, 1.275 (0.954–1.705) (*P* = 0.605). After adjusting for multiple variables (Table 2), the ORs of the 1^st^ quintile was also larger than the OR of the 5^th^ quintile with marginal significance (*P* = 0.067). The trend of ORs for anemia was downward according to the serum 25(OH)D quintiles (*P*_trend_ = 0.014). However, when the logistic regression analysis was conducted after the stratification by sex and menstrual status, the significance of OR trend was shown only in the female group. The predicted probability of anemia with 25(OH)D, after full adjustment, is shown graphically in figure 3. The predicted probability of anemia increased non-linearly with decreasing serum 25(OH)D concentrations, particularly at certain concentrations of 25(OH)D in females. However, there was no obvious trend in males. Among the covariates, there were no important interactions with serum 25(OH)D for the relationship with anemia.

**Table 2:** Adjusted odds ratios for anemia according to the serum 25-hydroxyvitamin D levels.

Quintiles	Range (ng/mL)	OR (95% CI)*	*P*
Total (n = 11,206)			0.014†
1^st^ quintile (n = 2,242)	<12.47	1.387 (0.978–1.968)	0.067
2^nd^ quintile (n = 2,237)	12.47–15.43	1.144 (0.816–1.602)	0.434
3^rd^ quintile (n = 2,248)	15.44–18.27	1.128 (0.796–1.598)	0.496
4^th^ quintile (n = 2,239)	18.28–22.37	0.867 (0.602–1.248)	0.440
5^th^ quintile (n = 2,240)	>22.37	1 (Reference)	
Male (n = 4,912)			0.532†
1^st^ quintile (n = 982)	<13.74	0.631 (0.301–1.322)	0.222
2^nd^ quintile (n = 986)	13.74–16.87	0.990 (0.522–1.877)	0.975
3^rd^ quintile (n = 979)	16.88–19.81	0.584 (0.294–1.159)	0.124
4^th^ quintile (n = 982)	19.82–23.89	0.547 (0.253–1.182)	0.124
5^th^ quintile (n = 983)	>23.89	1 (Reference)	
Premenopausal female (n = 3,130)			0.016†
1^st^ quintile (n = 627)	<11.12	1.757 (1.027–3.006)	0.040
2^nd^ quintile (n = 624)	11.12–13.51	1.625 (0.974–2.711)	0.063
3^rd^ quintile (n = 628)	13.52–15.89	1.477 (0.872–2.501)	0.146
4^th^ quintile (n = 624)	15.90–18.97	1.161 (0.719–1.874)	0.540
5^th^ quintile (n = 627)	>18.97	1 (Reference)	
Postmenopausal female (n = 3,164)			0.007†
1^st^ quintile (n = 632)	<12.46	1.927 (1.205–3.080)	0.006
2^nd^ quintile (n = 633)	12.46–15.59	1.144 (0.711–1.841)	0.578
3^rd^ quintile (n = 633)	15.60–18.54	1.212 (0.730–2.013)	0.456
4^th^ quintile (n = 633)	18.55–22.96	1.042 (0.628–1.731)	0.872
5^th^ quintile (n = 633)	>22.96	1 (Reference)	

* Adjusted for age, sex, smoking, exercise, hypertension, diabetes mellitus, cardiovascular disease, body mass index, season, laboratory findings such as ferritin, iron, total iron-binding capacity, cholesterol, triglyceride, estimated glomerular filtration rate, and daily iron intake.

† *P* value for trend.

Abbreviations: OR, odds ratio; CI, confidence interval.

## Discussion

Anemia and vitamin D deficiency are both important health issues, but their potential relationship remains less established in the general population. In this population-based study using data from a nationally representative survey, we first demonstrated a non-linear relationship between serum 25(OH)D and hemoglobin levels, and the levels of hemoglobin began to decrease below a 25(OH)D threshold of 26.4 ng/mL independently of other confounding factors. However, the threshold level was different according to the sex and menstrual status as follows: males, 27.4 ng/mL; premenopausal females, 11.8 ng/mL; postmenopausal females, 13.4 ng/mL. Additionally, the threshold level affected the pattern of association between serum 25(OH)D levels and the risk of anemia: the anemia risk increased as the serum 25(OH)D level decreased, but the trend was not linear in females irrespective of menstrual status. However, the correlation between 25(OH)D and anemia was not significant in males, although a threshold level was obtained from the GAM analysis. The present study raises important issues, first of all, providing vitamin D as a potential therapeutic option for the management or prevention of anemia.

Vitamin D has favorable pleiotropic actions beyond its pivotal role in calcium homeostasis and bone metabolism [27]. Indeed, previous clinical studies have shown that the risks of various non-skeletal diseases and mortality increase as serum 25(OH)D levels decrease [28]. Recently, growing evidence has suggested that this inverse association may be non-linear, in which the risks of clinical consequences related to vitamin D deficiency increase proportionally as 25(OH)D levels decrease below a certain threshold [16], [19], [20]. The identification of 25(OH)D thresholds according to an individual disease may contribute to establishing vitamin D replacement strategies by providing disease-specific target levels of vitamin D and identifying the proportion of a specific population with a potential risk of the disease. A meta-analysis reported that cardiovascular risk increased monotonically across decreasing 25(OH)D concentrations below approximately 24 ng/mL using a fractional polynomial spline regression model [19]. Another study reported that a composite outcome of incident hip fracture, myocardial infarction, cancer, and mortality increased below 22 ng/mL [20]. In this regard, vitamin D may also have a threshold effect on other clinical consequences, including anemia. Although the potential association between vitamin D and anemia has been suggested, all previous studies have focused on the correlation in population subgroups, such as those with chronic kidney disease, diabetes mellitus, heart failure, and elderly subjects [8], [9], [10], [11], [12], [13], [14]. In addition, the patterns of their associations have not been comprehensively evaluated, even in these subgroups. One previous study was conducted in the general population, but that study did not also consider the non-linear relationship between vitamin D and hemoglobin levels [15]. In the present study, we evaluated the pattern of association between vitamin D and hemoglobin levels over a wide range of 25(OH)D levels in a representative Korean adult population. There was a non-linear relationship with a threshold effect between serum 25(OH)D and hemoglobin levels. A substantial proportion of study subjects had a 25(OH)D level below the threshold defined in the present study; this may lead to clinical or subclinical anemia in study subjects. According to the NHANES data [29], the prevalence of vitamin D deficiency is different across racial/ethnic subgroups: particularly high in non-Whites. It is well known that Asians (especially those living in countries north of the equator) are at greater risk of developing vitamin D deficiency [30]. Therefore, the study findings may have an important implication especially in Asians and help to provide an optimal level of vitamin D that may potentially confer additional benefit for the correction of anemia, although randomized controlled trial remains elusive.

The non-linear trend was different according to sex and menstrual status, although the reason for this difference was not fully understood. There was no obvious trend between vitamin D and anemia in males, although a threshold level was obtained from the GAM. This sex-difference in correlation was also shown in other studies [15]. The level of sex-hormone such as estrogen may be related to the sex-difference in the correlation [31]. Furthermore, the sex-difference has been documented in anemia-related disease other than vitamin D deficiency [32]. Menstrual status is related to the anemia risk. In general, premenopausal females may have a risk of iron-deficiency anemia due to menstruation, while postmenopausal females may have risks of both nutritional deficiency or anemia of inflammation due to old age. If the iron status was not adjusted in a GAM model (data not shown), the threshold levels were 25.6 ng/mL in premenopausal females (11.8 ng/mL after adjustment) and 13.4 ng/mL in postmenopausal females (13.4 ng/mL after adjustment), respectively. This result suggests that iron status (iron deficiency anemia) has a greater effect on the correlation in premenopausal females than in postmenopausal females. Additionally, other factors such as hormonal effect may be also related to the difference between premenopausal and postmenopausal females. However, this issue in premenopausal females does not diminish the importance of the threshold effect of vitamin D on hemoglobin level because of their steep relationship below the threshold.

Vitamin D supplementation has long been known to improve anemia and reduce the need for erythropoietin in dialysis patients [33], [34]. However, the underlying mechanism has not been established to date. It is suggested that vitamin D plays a role in the suppression of the inflammatory milieu that contributes to the development of anemia. The reduction of inflammatory cytokines after vitamin D supplementation in some experimental studies supports this possibility [35], [36]. An observational study also supports this mechanism, demonstrating that low vitamin D is associated with a high risk of “anemia of inflammation” [13]. A direct effect of vitamin D on erythroid precursors is also possible. Vitamin D in hematopoietic tissues affects the proliferation of erythroid precursor cells via increased calcium permeability [37] or increased erythropoietin receptor expression [38]. Furthermore, vitamin D can affect hematopoietic tissue in a paracrine fashion because the vitamin D receptor is also expressed in bone marrow [39]. Future experimental studies are needed to establish the underlying mechanisms of the present cross-sectional study results.

The present study used a dataset from the KNHANES, a study conducted in a representative sample of the general Korean population under strict quality control criteria. Although the present study has important strengths, it also has some limitations. First, reverse causation cannot be excluded as an explanation for our results because of the cross-sectional study design. However, the biological plausibility suggested by experimental studies supports a causal link [37], [38], [39]. Furthermore, the improvement in anemia after vitamin D supplementation has been observed in prospective studies of dialysis patients [33], [34], making reverse causation less likely. Nevertheless, further randomized clinical trials are needed to elucidate whether the hemoglobin level or the risk of anemia is modified by vitamin D replacement therapy. Second, data on the confounding factors such as status of vitamin D absorption were not available in KNHANES. Third, we conducted this study only in Asian adults. Therefore, care must be taken when applying our study results to other ethnicities.

In conclusion, we first confirmed the non-linear relationship between serum 25(OH)D and hemoglobin levels and the threshold for this association in Korean females. The present findings should be considered in future clinical studies addressing vitamin D and hemoglobin. Additionally, further clinical and experimental studies may be warranted to validate the present findings and determine whether the correction of vitamin D deficiency can ameliorate anemia.
